# Assessing METland^®^ Design and Performance Through LCA: Techno-Environmental Study With Multifunctional Unit Perspective

**DOI:** 10.3389/fmicb.2021.652173

**Published:** 2021-06-11

**Authors:** Lorena Peñacoba-Antona, Jorge Senán-Salinas, Arantxa Aguirre-Sierra, Pedro Letón, Juan José Salas, Eloy García-Calvo, Abraham Esteve-Núñez

**Affiliations:** ^1^METfilter S.L., Seville, Spain; ^2^IMDEA Water Institute, Parque Científico Tecnológico, Madrid, Spain; ^3^Foundation Centre for New Water Technologies (CENTA), Seville, Spain; ^4^Department of Analytical Chemistry and Chemical Engineering, University of Alcalá, Madrid, Spain

**Keywords:** life cycle assessment, METland, Net Environmental Balance, Funtional Unit, wastewater treatment, treatment wetlands, constructed wetland, principal component analysis

## Abstract

Conventional wastewater treatment technologies are costly and energy demanding; such issues are especially remarkable when small communities have to clean up their pollutants. In response to these requirements, a new variety of nature-based solution, so-called METland^®^, has been recently develop by using concepts from Microbial Electrochemical Technologies (MET) to outperform classical constructed wetland regarding wastewater treatment. Thus, the current study evaluates two operation modes (aerobic and aerobic–anoxic) of a full-scale METland^®^, including a Life Cycle Assessment (LCA) conducted under a Net Environmental Balance perspective. Moreover, a combined technical and environmental analysis using a Net Eutrophication Balance (NEuB) focus concluded that the downflow (aerobic) mode achieved the highest removal rates for both organic pollutant and nitrogen, and it was revealed as the most environmentally friendly design. Actually, aerobic configuration outperformed anaero/aero-mixed mode in a fold-range from 9 to 30%. LCA was indeed recalculated under diverse Functional Units (FU) to determine the influence of each FU in the impacts. Furthermore, in comparison with constructed wetland, METland^®^ showed a remarkable increase in wastewater treatment capacity per surface area (0.6 m^2^/pe) without using external energy. Specifically, these results suggest that aerobic–anoxic configuration could be more environmentally friendly under specific situations where high N removal is required. The removal rates achieved demonstrated a robust adaptation to influent variations, revealing a removal average of 92% of Biology Oxygen Demand (BOD), 90% of Total Suspended Solids (TSS), 40% of total nitrogen (TN), and 30% of total phosphorus (TP). Moreover, regarding the global warming category, the overall impact was 75% lower compared to other conventional treatments like activated sludge. In conclusion, the LCA revealed that METland^®^ appears as ideal solution for rural areas, considering the low energy requirements and high efficiency to remove organic pollutants, nitrogen, and phosphates from urban wastewater.

## Introduction

Nowadays, one of the main environmental problems is water scarcity and ecological degradation of the water body status (UNESCO, 2018). Indeed, it was estimated that by 2015, still 2.3 billion people in the world would not have access to basic sanitation facilities (UNICEF and WHO, 2017). Although Wastewater Treatment (WWT) systems have improved in recent years, the optimization and implementation in all the populations remain as a high priority. However, small communities and isolated houses have no access to well-functioning sanitation infrastructure due to limited technical and economic resources. It is therefore proposed that WWT should be low-cost technologies with simple operation and maintenance (Mahmood et al., 2013). In order to protect the environment in these situations, a number of decentralized technologies, so-called Nature Based Solutions (NBS), have been developed based on eco-efficiency designs with low operational expenses (constructed wetlands, stabilization ponds, and sand filters, etc.) (Massoud et al., 2009).

In this eco-design context, METland^®^ is an innovative nature-based solution that merges Microbial Electrochemical Technologies (MET) with Constructed Wetlands (CW). The main constructive difference resides in replacing the classical biofiltering material (gravel, sand) of CWs by electroconductive (EC) granular material. This EC material allows the electrons to circulate through the material, avoiding the classical electron acceptor limitation from anoxic environments, so METland^®^ operates maximizing the electron transfer between the EC material and the electroactive bacteria (Rotaru et al., 2021). Thus, METland^®^ is a term to denominate such general concept and it does not imply any specific operation mode. So, such systems can be operated either under flooded and anoxic mode (Aguirre-Sierra et al., 2016) or under downflow aerobic one (Aguirre-Sierra et al., 2020). Interestingly, bacteria from *Geobacter* genus were abundant as part of the electroactive biofilm regardless of the operation, anaerobic or aerobic (Aguirre-Sierra et al., 2016, 2020). Probably the most relevant consequence of stimulating the electroactive microbial communities from METland^®^ was a vast enhancement of biodegradation rates and consequently a feasible reduction of the footprint requirements (Wang et al., 2020). Moreover, this technology is a suitable on-site solution for the treatment of WW, including micropollutants (Pun et al., 2019), with the clear advantage of no energy consumption or sludge generation (Ramírez-Vargas et al., 2019). A deep exploration of materials and design was carried out in the frame of the iMETland project^1^, which aimed to implement such innovative solution for cleaning up WW from two small communities (200 p.e.) at Spain and Denmark with a ratio of 0.4 m^2^/pe. The availability of materials for constructing such MET-based solutions can be a drawback for reaching a global implementation; however, recent studies have explored such issues through a circular economy approach to reveal how the physicochemical properties of carbonaceous material (e.g., EC coke and EC biochar) correlates with the biodegradation of pollutant (Prado et al., 2019). Regarding innovative designs, the use of the so-called e-sinks devices was proved to effectively control the electron flow inside the EC bed for enhancing removal rates of pollutants (Prado et al., 2020). Prediction tools for finding suitable locations to implement METland^®^ have been recently developed through a methodology based on Multi-Criteria Evaluation (MCE) techniques and Geographical Information Systems (GIS) (Peñacoba-Antona et al., 2021). The interest and potential of the solution seems clear but, in the context of a Circular Economy transition, the potential environmental impact of a new technology like METland^®^ should be evaluated to prevent impacts from the design (EEA, 2019).

The Life Cycle Assessment (LCA) methodology is a standardized methodology to quantify the environmental impact associated with a system or product (ISO 14040, 2006; ISO 14044, 2006). LCA allows the introduction of different life-cycle stages from the building to the demolition phase, and enables better decision making due to the inclusion of the quantification of the effects of the entire system under study (Lundin et al., 2000). Therefore, LCA fits as a decision-making tool for technological environmental assessment under the Circular Economy framework (Zhao et al., 2019).

Among all review publications regarding LCA applied to WWT technologies, Corominas et al. (2013) is probably one of the most extensive and complete study covering such topic. This review analyzed the variability and the lack of consensus in the methodological choices within the LCA studies, including phases, scope and goal definition, as well as the boundary selection. Highlighting the necessity to develop standardized guidelines for WWT more detailed than ISO 14040 and ISO 14044 (2006) and Corominas et al. (2013) pointed out the selection of the Functional Unit (FU) as one of the most critical points. Furthermore, this fact has been also mentioned in other reviews, indicating the difficulties to compare the LCA studies because of the discordance in the FU (Zang et al., 2015) or the vague definition (Gallego-Schmid and Tarpani, 2019). Other criticisms associated with the selection of the FU were made in relation to the lack of representativeness, for example, 1 m^3^ of wastewater (it does not include the effectiveness) (Godin et al., 2012; Corominas et al., 2013; Lorenzo-Toja et al., 2018), population equivalent (country-based differences), grams of phosphorus (organic material not included), or COD equivalent (non-C-based pollutants not considered) (Zhu et al., 2013; Niero et al., 2014). Aside from that, there is a main tendency in WWTs to consider a volumetric FU. Indeed, there is a general consensus to consider the treatment of 1 m^3^ of WW as FU (Corominas et al., 2013; Gallego-Schmid and Tarpani, 2019).

Focusing on the environmental impact of decentralized systems, some LCAs were performed comparing alternative WWT processes for small and rural communities (Machado et al., 2007; De Feo and Ferrara, 2017; Arashiro et al., 2018). These studies concluded that nature-based solutions like CWs had a lower impact compared with conventional systems like aeration activated sludge due to the high energy consumption of the last (Garfí et al., 2017). Otherwise, previous LCA studies on CWs pointed out the capacity of the system to couple with different flow mode and loadings rates with low energy and no chemical requirements (Lutterbeck et al., 2017; Flores et al., 2019). Additionally, some alternatives to the conventional CW had included artificial aeration (Resende et al., 2019). An LCA of a variety of CW integrating single electrodes for harvesting energy was studied by Corbella et al. (2017), revealing lower environmental impact than a conventional CW with a notably reduction of volume but a higher construction cost.

The objective of this study was the techno-environmental comparison of two different conceptual designs of METland operating at full scale with real urban wastewater, including a multifunctional unit (MFU) study performed to increase the accuracy of future decisions.

## Materials and Methods

### Designs and Operational Description

**FIGURE 1:**
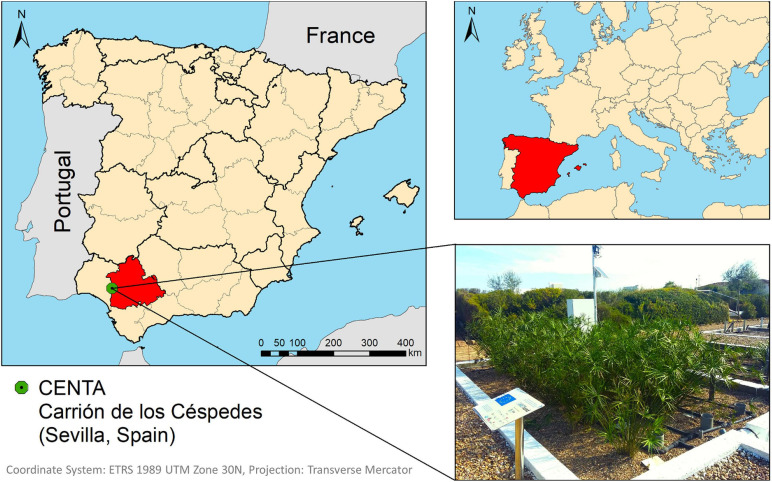
Location of the METland^®^ unit at Municipal WWT plant (Carrión de los Céspedes, Spain).

**FIGURE 2:**
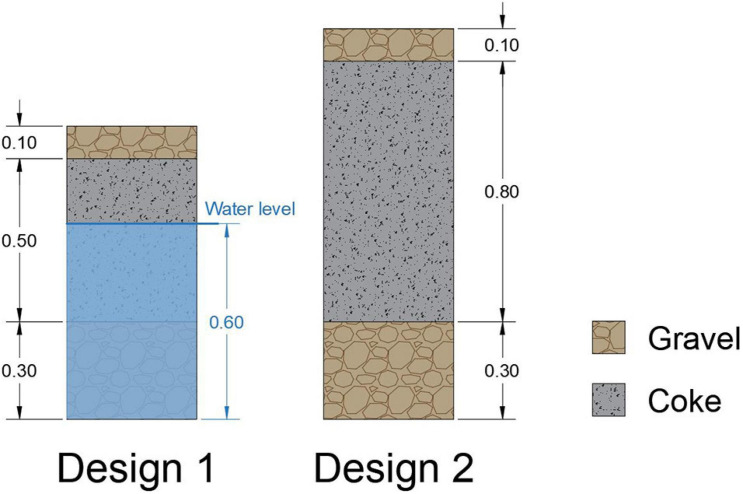
Construction profiles of METland^®^ designs validated in this work.

A METland^®^ unit was built in the facilities of Foundation Centre for New Water Technologies (CENTA) for testing the technology with real WW (Figure 1). This METland^®^ unit was operated in two different configurations. Firstly, design 1 (D1) was constructed with the following vessel dimensions: 6.5 m length, 3.7 m width, and 1.2 m depth within a surface area of 24 m^2^. The bed material was divided into three layers with different thicknesses and materials (Figure 2).

i. A bottom layer (0.3 m of river gravel) to create a volume of rounded material for conducting the water to the drainage system as well as incorporate the pipes.
ii. An intermediate layer (0.5 m of EC material) to generate a favorable environment for the growth of bacterial communities, specially EC species from *Geobacter* genus, previously reported (Aguirre-Sierra et al., 2016).
iii. A top layer (0.1 m of gravel) to isolate the system from direct sun radiation. In addition, it attenuates the temperature variations inside the system.

Secondly, the previous design was modified into a second design (D2). Constructed differences were strictly based on the increasing of thickness of the intermediate layer (from 0.5 m to 0.8 m) of the conductive material. The characteristics of METland^®^ designs (D1 and D2) are shown in Figure 2.

On top of the differences in design, each configuration kept its own operational mode. In D1, the water level reached 60 cm by flooding completely the bottom layer and partially the intermediate-conductive layer. Thus, D1 was partly operated under aerobic and anaerobic conditions. Contrarily, D2 operated strictly under aerobic conditions by percolating WW pulses through the layers till finally water was collected through the drainage system.

Both designs were operated under different COD loading rates and flow rates according to Table 1. D1 was assayed in two independent and consecutive periods with different organic loading rate (Biochemical Oxygen Demand, g BOD_5_ m^–3^): first, a medium loading rate period (P1), followed by a high loading rate one (P2). Moreover, D2 was assayed in three periods with increasing loading rate (P3, P4, and P5, respectively).

**TABLE 1:** Summary of designs.

**System characteristics**					**Period**		**Inlet loadings (average)**		
**Design**	**Flow mode**	**Metabolism**	**^*EC*^Coke bed thickness (m)**	**^*EC*^Coke bed volume (m^3^)**	Code*	**Days**	**Flow rate (m^3^⋅d**^–^**^1^)**	**Total flow (m^3^)**	**COD (g⋅m**^–^**^3^)**
**Design 1**	Vertical- partially water saturated	Combination (aerobic /anaerobic)	0.5	12.02	**D1**	196	2.7	528	4.67E+02
									(± 48%)
					**P1**	107	2.6	276	3.82E+02
									(± 30%)
					**P2**	89	3	252	5.59E+02
									(± 52%)

**Design 2**	Vertical-unsaturated	Aerobic	0.8	19.24	**D2**	247	5.59	1290	3.82E+02
									(± 30%)
					**P3**	71	5.4	290	2.31E+02
									(± 58%)
					**P4**	22	5.4	120	2.95E+02
									(± 9%)
					**P5**	154	5.9	877	4.44E+02
									(± 15%)

*Codes: Design (D) and flow rate-based operation mode (P) (between parenthesis in the inlet quality parameters the variation coefficient in percentage).

### Evaluation of METland^®^ Performance

The system was fed under discontinuous flow mode with urban WW (post-primary treatment) from the municipality of Carrión de los Céspedes (2,500 inhabitants in 2018; Seville, Spain). METland performance was characterized by weekly sampling from both influent and effluent of the system. The analysis was performed following the standard methods for Total Suspended Solids (TSS), Biochemical Oxygen Demand (BOD_5_), Chemical Oxygen Demand (COD), Total Nitrogen (TN), and Total Phosphorus (TP) (American Public Health Association, 2005). The loading rates and the removal efficiency were obtained using the weighted average for the calculation of the influent and removal rates through the different periods. The loading rates were calculated for BOD_5_, TSS, N, and P fed in the METland^®^ with respect to a volumetric unit of bed and day. Such parameter considers the variations in flow mode and bed volume during the different periods, allowing to normalize results. Thus, a two-way analysis of variance was conducted to test the data statistical significance, integrating the effect of influent fluctuations within removals in each design (two-way ANOVA). The comparison among means was tested using R (R Core Team, 2018) with a significance level of *p*-value < 0.05 (95% confidence).

In relation to the effluent concentration of pollutants, the European Union establishes a limit for being able to discharge the water into the environment (EEC, 1991). Particularly, for small agglomerations, the Council Directive 91/271/EEC imposes a limit for the main parameters of the quality of water (BOD_5_, COD, TN, TP, and TSS). Furthermore, a correlation was performed between those parameters (influent concentration (I), effluent concentration (O), and effectiveness (E-in percentage of removal)) using R (R Core Team, 2018).

### Life Cycle Assessment (LCA)

The full analysis for the selection of the best design should include the environmental impacts associated with the technology. An LCA was performed as an environmental management technique developed to address these impacts. The LCA methodology was applied following the four phases described in ISO 14040 and ISO 14044 (2006): (i) the goal and scope definition, (ii) the inventory analysis, (iii) the impact assessment, and (iv) the interpretation. Additionally, the study was developed in accordance to the International Reference Life Cycle Data System (ILCD) technical guidance (JCR, 2010).

### Goal and Scope Definition

The goal of the present study was to select the most environmentally friendly design among two independent METland^®^ configurations. An attributional LCA study was performed along the construction and operation phases, including the monitoring. Coherently with the effectiveness analysis, the scenarios in the LCA compared two designs: D1 and D2, as well as the differences or particularities within several operation periods: P1, P2, P3, P4, and P5 (described in section “Designs and Operational Description”).

To this aim, 1 m^3^ of treated WW was defined as FU. However, such FU does not include information about chemical nature of WW (e.g., BOD_5_). These considerations are important for testing systems with real WW because this medium is variable so systems will never be tested with WW of identical composition. Therefore, a MFU approach was also conducted (fully described in section “Multi-Funtional Unit Assessment”).

**FIGURE 3:**
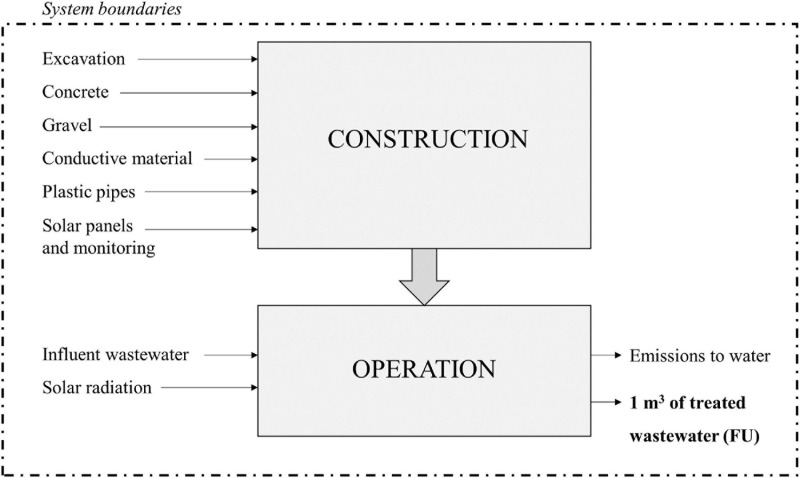
System boundaries for the LCA of the METland^®^ designs.

The system boundaries include the processes related to the construction and operation over a 25-year horizon period (Figure 3). The dismantling phase was considered non-significant in relation to the complete analysis and excluded. Additionally, all stages were systematically studied regarding both input and output flows of materials, energy, and intermediate processes. The final effluent direct emissions were also considered. Emissions to air such as direct Green House Gases (GHGs) were not accounted in this study due to the lack of such on-site data for our METland^®^ systems.

### Inventory Analysis

The results of the inventory of construction phase are summarized in Table 2, divided in the two generic designs and five periods. The lifespan of the construction for the inventory was assumed to be ca. 25 years. This assumption (ca. 15–30 years) is within the range previously reported by CW literature (Corominas et al., 2013; Lopsik, 2013; Garfí et al., 2017). Furthermore, this METland^®^ unit was performed reusing the construction of a previous peat filter. Thus, in a new construction, the concrete will be replaced by a geotextile, reducing the overall environmental impact.

**TABLE 2:** METland^®^ construction inventory.

	**Units**	**Designs**						
		
		**D1**	**P1**	**P2**	**D2**	**P3**	**P4**	**P5**
**Construction**								
Excavation	m^3^⋅m^–3^	1.45E-03	1.52E-03	1.29E-03	6.99E-04	7.29E-04	7.20E-04	6.65E-04
Concrete	m^3^⋅m^–3^	2.12E-04	2.21E-04	1.88E-04	1.02E-04	1.06E-04	1.05E-04	9.70E-05
Gravel	m^3^⋅m^–3^	3.91E-04	4.09E-04	3.47E-04	1.88E-04	1.96E-04	1.94E-04	1.79E-04
Coke	m^3^⋅m^–3^	4.89E-04	5.11E-04	4.34E-04	3.77E-04	3.93E-04	3.88E-04	3.58E-04
**Pipes**								
- PE*	kg⋅m^–3^	4.28E-04	4.47E-04	3.79E-04	2.06E-04	2.15E-04	2.12E-04	1.96E-04
- PVC*	kg⋅m^–3^	1.78E-03	1.86E-03	1.58E-03	8.57E-04	8.93E-04	8.82E-04	8.14E-04
- Injection	kg⋅m^–3^	5.08E-04	5.31E-04	4.51E-04	2.45E-04	2.55E-04	2.52E-04	2.33E-04
- Extrusion	kg⋅m^–3^	1.70E-03	1.77E-03	1.51E-03	8.18E-04	8.52E-04	8.43E-04	7.78E-04
**Monitoring**								
- Photovoltaic single-Si panel	m^2^⋅m^–3^	5.99E-06	6.25E-06	5.31E-06	2.88E-06	3.00E-06	2.97E-06	2.74E-06

Values are referred to the FU (1 m^3^ of treated WW). *PVC (Polyvinyl chloride) and PE (Polyethylene).

The operation inventory accounts the water quality related just to the performance of the METland unit (without the primary treatment) and monitoring (summarized in Supplementary Table A1). The energy needed for monitoring was obtained from solar radiation using photovoltaic panels. In CENTA, the radiation rate in the period analyzed was 18.54 MJ/m^2^, according to the Spanish Agency of Meteorology (AEMET, 2020).

### Life Cycle Impact Assessment Method

The potential environmental impact of METland^®^ designs was calculated using the software OpenLCAv1.8 (OpenLCA.org., 2006). Background processes were obtained from Ecoinvent3.4 database (Wernet et al., 2016), summarized in Supplementary Table A2. The impact method selected was Hierarchical ReCiPe Midpoint (Goedkoop et al., 2013). Table 3 summarizes the 10 impact categories and their abbreviators. The impact categories have been chosen following the tendency observed in the literature (Godin et al., 2012; Garfí et al., 2017; Flores et al., 2019).

**TABLE 3:** Selected impact categories from the impact method ReCiPe Midpoint (H).

**Code**	**Impact category**	**Reference unit**
CC	Global warming potential	kg CO_2_ eq.
OD	Ozone depletion potential	kg CFC-11 eq.
FE	Freshwater eutrophication potential	kg P eq.
ME	Marine eutrophication potential	kg N eq.
HT	Human toxicity potential	kg 1,4-DCB eq.
POF	Photochemical oxidant formation potential	kg NMVOC
PMF	Particulate matter formation potential	kg PM_10_ eq.
FET	Freshwater ecotoxicity potential	kg 1,4-DCB eq.
METP	Marine ecotoxicity potential	kg 1,4-DCB eq.
FD	Fossil depletion potential	kg oil eq.

### Net Eutrophication Balance

Net Environmental Balance (NEB) perspective proposed by Godin et al. (2012) and Igos et al. (2012) allows to take into account the difference between discharging the WW directly into the environment (null option) or treating the WW (WWT scenario). However, this perspective inspired the development of indicators such as Eutrophication Net Environmental Impact (ENEI) proposed by Lorenzo-Toja et al. (2016). Centering in the eutrophication category relativized the NEB by the distance to target goals of legislation in terms of eutrophication. These perspective and indicators allow the consideration into the analysis and results of the inlet qualities and treatment effectiveness and also represent better the environmental trades-offs of the technology. An important advantage fitted to the experimental conditions is described in section “Designs and Operational Description.”

Based on the precedent studies, in this analysis, we proposed an indicator focused on the water quality before and after the treatment. The Net Eutrophication Balance (NEuB) defined by Eq. 1 represents the impact avoided due to the removal of pollutants achieved in the WWT.

(Eq. 1) $$\text{NEuB}={\text{EuP}}_{\text{i}}-{\text{EuP}}_{\text{e}}-{\text{EuP}}_{\text{p}}$$

EuP_*i*_, eutrophication potential of the direct discharge of WW.
EuP_*e*_, eutrophication potential caused by the discharge of the treated effluent.
EuP_*p*_, indirect eutrophication potential produced by the WWT processes.

NEuB is similar to NEB performed by Godin et al. (2012) but, in our case, it was focused in the selected eutrophication categories of the ReCiPe-Midpoint (H): ME and FE. This method difference represents separately the eutrophication impact associated with the Nitrogen (N) or Phosphorus (P) emissions, respectively.

## Results and Discussion

METland^®^ are solutions for effectively removing pollutants from WW. Interrogating the technology through a Life Cycle Assessment would provide info about its sustainability. Thus, in the current section, we will present and discuss the results from a techno-environmental analysis and a MFU study regarding different conceptual designs like mixed (aerobic/anaerobic) from D1 to fully aerobic D2.

### Effectiveness Analysis

**FIGURE 4:**
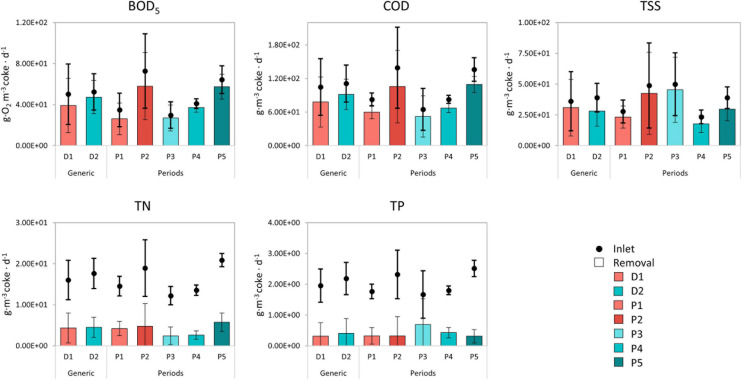
Pollutant removal rates normalized per cubic meter of bed material for both designs (D1 and D2) and five periods (P1–P5). The data correspond to the METland^®^ unit itself (without considering the primary treatment). Columns so-called *generic* were calculated as the average of different periods. Error bars represent the standard deviation.

Our METland^®^ units were treating real urban WW after a primary sedimentation of solids, and fulfilling the WWT discharge limits (EEC, 1991) for all conditions tested and regarding the population and the vulnerability of the implementation area. Removal rates of pollutants must be normalized per unit of biofiltering material (m^3^) in order to validate the efficiency of the technology under different scenarios (Figure 4).

Regarding organic pollutants present in WW, the most important indicators are COD and BOD_5_, and indeed, both parameters revealed a higher removal in D2 than D1. There were significant statistical differences (*p* < 0.05) between D1 and D2 with a *p*-value of 4.4⋅10^–4^ for COD and 1.3⋅10^–6^ for BOD_5_. Precisely, the mixed aerobic–anaerobic nature of D1 removed 90% BOD_5_, while the purely aerobic D2 unit removed 94% of BOD_5_ regarding the raw WW. The results were consistent with data obtained in previous studies of METland^®^ configurations (Prado et al., 2019; Ramírez-Vargas et al., 2019), including those reported by Aguirre-Sierra et al. (2020) with COD removal ranging from 82 to 99% in vertical down-flow configuration. The differences were possibly related with the higher oxygen availability due to the passive aeration of the downflow (D2) configuration. This situation is similar to the one found in constructed wetland operated under vertical flow. The removal rate per cubic meter of bed material was ca. 80 g COD per m^3^ of bed per day for D1 and ca. 90 g COD per m^3^ of bed⋅d^–1^ for D2 under the tested conditions. The periods analyzed correspond to first stages of operation; however, other studies using mature systems showed removal rates in the range of 150–200 g COD per m^3^ of bed per day (Aguirre-Sierra et al., 2017). These results suggest a similar removal rate than the ones achieved in additional experiences with one step EC bed where unsaturated and saturated zones co-exist in the same system (Cabred et al., 2019).

The biofiltering nature of METland^®^ also exhibits a vast removal of those fine solids not properly removed by primary treatments. No significant differences were identified between models regarding TSS removal (*p* < 0.05). Thus, probably due to the higher hydraulic retention time of anoxic step in D1, such mixed aerobic–anaerobic design slightly outperformed D2 by 5% in terms of TSS removal efficiency, achieving 90% of average removal within the WWT. Similar results were reported by the Ramírez-Vargas et al. (2019) analysis with MET-CW-based anaerobic columns, 85–90% of TSS removal. Furthermore, the low growth yield typically detected in electroactive microorganism like Geobacter counteracts any clogging issue inside the bed (Mahadevan et al., 2006). In contrast, nitrogen removal on METland^®^ is performed in two phases: nitrification that occurs under aerobic conditions (oxidation of ammonia to nitrate) and denitrification, reduction from nitrate to nitrogen gas, typically enhanced under anaerobic conditions (Aguirre-Sierra et al., 2017, 2020). Under aerobic–anoxic configuration (D1), both processes were feasible due to the anaerobic environments present in the inner part of the biofilm where *Geobacter* genus was detected (Aguirre-Sierra et al., 2020). Actually, D1 achieved stable removal rates in response to a variable loading, with an average reduction of 26% (4.53 g TN m^–3^ ⋅d^–1^). In contrast, D2 seems more influenced by the oxygen availability and, consequently, the ammonia removal was higher than in D1 via nitrification, revealing significant differences among designs (*p*-value = 1.1⋅10^–9^). Interestingly, both configurations show a similar behavior in TN removal (*p*-value = 0.07), suggesting an unexpected denitrification step even under passive aeration from D2. Such denitrification is supported by the presence of anoxic environments in the inner part of the biofilm where *Geobacter* was detected through microbial community analysis (Aguirre-Sierra et al., 2020).

In terms of TP, the removal efficiency ranged between 12% (P5) and 41% (P3). Both designs revealed a decrease in the removal of TP correlated with the increase of flow rate, for example, in D1, the removal decreased from 18% in P1 to 14% in P2. Thus, the removal processes were similar to those typically found in constructed wetland, mainly due to de-adsorption by the bed substrate (Bolton et al., 2019). In D1, the volume of EC material was 60% less in comparison to D2, but D2 periods present two times higher flow rate. D1 exhibited 16% average removal of TP compared to 18% in D2. Furthermore, if the primary treatment is included, the removal rate of TP increased to 36% in D1 and 20% in D2; this difference among designs could be due to the retention time. Nevertheless, new EC materials based on biochar with capacity for removing nutrients are currently under investigation (Schievano et al., 2019) and, eventually, will lead to a new generation of METland^®^ where bed material may be fully recyclable at its end-of-life as a soil amendment.

**FIGURE 5:**
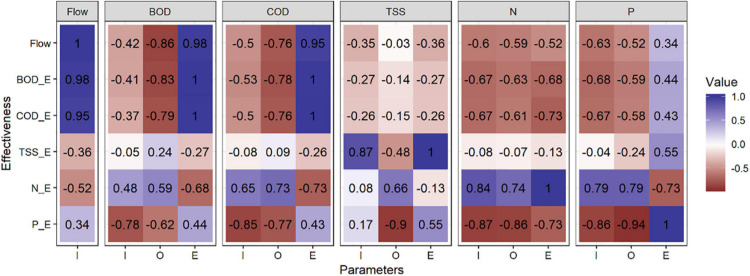
Correlation between the main parameters analyzed in the study and their effectiveness. Codes: Input (I), Output (O), and Effectiveness in % (E).

Our studies revealed how the removal efficiency of pollutants can be correlated with flow rate (Figure 5). In accordance to the literature, BOD_5_ and COD are indirect indicators of organic matter in urban WW (Hur et al., 2010). Indeed, a strong correlation (95–98%) between the COD–BOD_5_ effectiveness (COD_E and BOD_E, respectively) and the flow rate (m^3^ per day) was observed, mainly due to the direct relation between the loading rate and the concentration of organic matter per cubic meter. On the other hand, nitrogen (N_E) and phosphorus removal effectiveness (P_E) had an inverse correlation between them (−73%), because the mechanisms of P removal are related mainly to physical processes and the N removal is mainly due to biological ones (Kadlec and Wallace, 2008).

### Life Cycle Assessment Results

**FIGURE 6:**
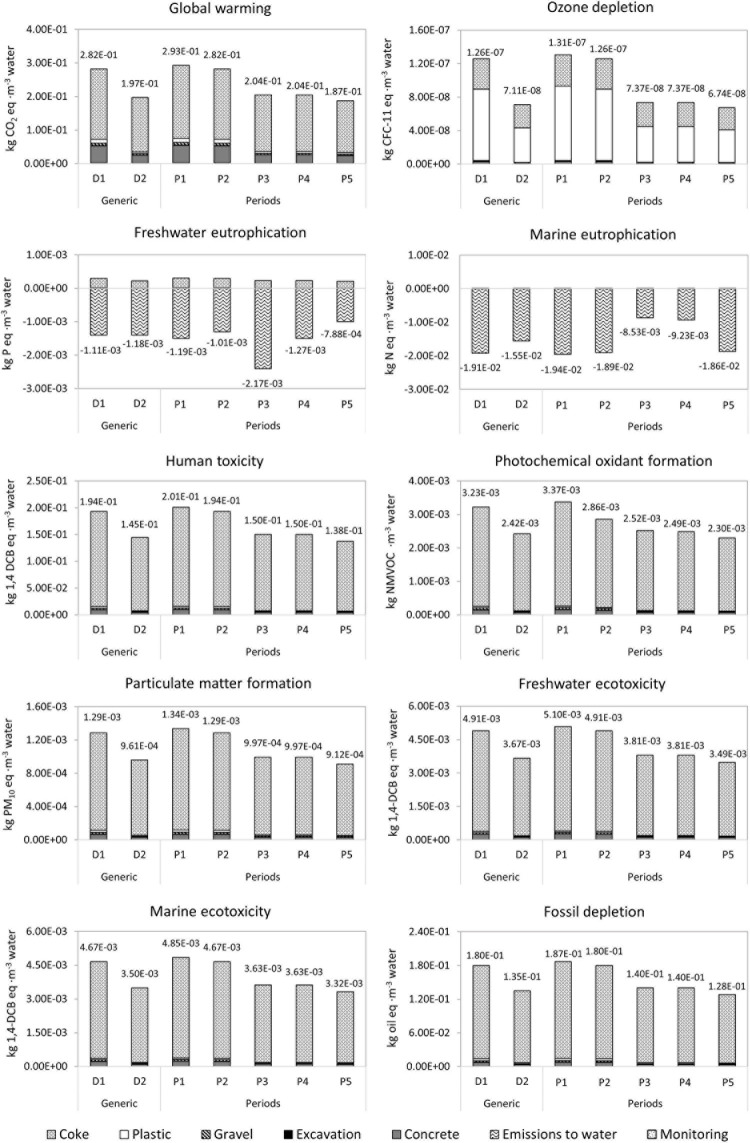
Potential environmental impacts and process contribution for the periods analyzed.

The results of the selected impact categories and the process contribution for each design and period are summarized in Figure 6. All the categories except the eutrophication ones (ME and FE) follow the same pattern and similar performances between design and periods. The overall environmental impact of D1 was 33–77% higher than D2. Interestingly, from P1 to P5, a tendency for the total impact to progressively decrease between periods could be noticed, probably due to the increase of the daily flow rate. This mathematical relationship strengthens the need for the MFU analysis. The cause is the distribution of construction impact in a major amount of volume treated during the service life of the WWT. Nonetheless, the NEuB perspective reduces the WWT influence on the eutrophication categories. In those categories, the balance of the impacts achieves a negative value for eutrophication, which represents the avoided impact associated with the N and P removal.

Additionally, the contribution of each process to the impact categories is presented in Figure 6. Construction phase presents a higher contribution in all the categories except for ME and FE, in which the operation phase was the most important one due to the avoided impact by the reduction of N and P emissions to water, mentioned before. A similar feature was found in the literature related to non-conventional technologies in which the environmental impacts are mainly influenced by the construction phase (Fuchs et al., 2011; DiMuro et al., 2014; Corbella et al., 2017; Lopes et al., 2020). This effect could be reasonably explained by the low energy and material flows associated with operation; indeed, METland^®^ does not require energy or chemical consumption and does not produce sludge since electroactive biofilm is tightly associated with the bed material. The unique energy flow included in the assessment was produced by those solar panels feeding the monitoring system (e.g., bioelectrochemical sensors). The energy savings of the system were mainly related to the absence of artificial aeration typically found in standard intensive WWTs (Dixon et al., 2003). On the other hand, assuming the total construction environmental impact in both conventional constructed wetland and METland^®^, the last one was able to treat higher volume of wastewater per footprint (Aguirre-Sierra et al., 2016, 2020).

The contribution profiles are similar between most of the categories (CC, FD, FET, HT, ME, PMF, and POF): coke (95–74%), concrete (3–18%), and plastic pipes (1–4%). Concretely, in the CC category, coke use represents 74% of D1 and 82% of D2 overall impact. Coke upstream processes accumulate most of the impact, that is, mainly due to the high temperatures and the energy required for this transformation from coal to coke within the pyrolysis process (Liu and Yuan, 2016). It must be mentioned that the life span of coke is longer than other CW materials; therefore, the construction impact will be lower distributed in time. Further works in METland^®^ should focus on the use of low impact and ultra-conductive materials such as EC biochar (charcoal) (Prado et al., 2019; Schievano et al., 2019). Even the usage of valorized agricultural wastes pyrolysed could be an environmentally friendly alternative to consider.

Otherwise, OD category presents a different impact profile in which the plastic pipes accumulate most of the impact (58–68%), followed by coke (28–38%) and the concrete almost insignificant (2%). This dissimilarity was well described in the study of Corbella et al. (2017). In addition, from the OD, it can be clearly deduced that D1 produces a 77% higher impact than D2, presenting the largest difference in total impact between designs. Furthermore, by contrast, categories of ME and FE present a negative value of N and P removal, respectively. In this case, the NEuB perspective provides the inclusion of the effectiveness of METland^®^ treatment. For FE, the greater avoided impact is associated with D2 and for the ME with D1. During P3, the most important P removal (42%) is achieved, resulting in the lowest impact (−2.17⋅10^–3^ kg P eq. m^–3^) (Figure 4). The trend observed is a lower impact with higher removal rate in N and P.

The herein studied METland^®^ generated 0.29 kg CO_2_ eq.⋅m^–3^. Other authors who previously modeled the impact of the theoretical integration of bioelectrochemical elements predicted a similar impact (0.34 kg CO_2_ eq.⋅m^–3^) if a hypothetical anode (59 m^3^) and a cathode (245 m^3^) made of graphite were integrated in a constructed wetland (Corbella et al., 2017). So, METland^®^ impact seems to be in the same range than other nature-based solutions (DiMuro et al., 2014; Lutterbeck et al., 2017) and significantly lower than activated sludge systems 1.2 kg CO_2_ eq.⋅m^–3^ (Machado et al., 2007; Garfí et al., 2017).

From an environmental point of view, the aerobic configuration (D2) appeared as the best alternative (cubic meter of treated wastewater, FU). Particularly, the low impact of the third period (P5) pointed out the high capacity of the treatment for high flow rates and, consequently, a reduction of the impact. Although the EC material used in the D2 construction was increased 60% in respect of D1, its higher effectiveness resulted in a lower impact.

### Multi-Functional Unit Assessment

As mentioned before, volume units as a FU do not necessarily reflect the removal efficiency for all parameters monitored in WW (Comas Matas and Morera Carbonell, 2012; Corominas et al., 2013; Gallego-Schmid and Tarpani, 2019). Therefore, the present study incorporates a MFU analysis. Results were aggregated around a total of six FUs to represent the eco-effectiveness of different parameters: (i) the treatment of 1 m^3^, used for the central analysis, (ii) the removal of 1 g of TSS, (ii) the removal of 1 g of BOD, (iv) the removal of 1 g of COD, (v) the removal of 1 g of TN, and (vi) the removal of 1 g of TP. The methodological proposal aims to improve the robustness of the decision by solving the uncertainties associated with the influent loads and the associated effectivities. Furthermore, for a deeper understanding, LCA results and operational conditions were analyzed together with Principal Component Analyses (PCA) assessed with R (R Core Team, 2018).

### Comparative Results

**FIGURE 7:**
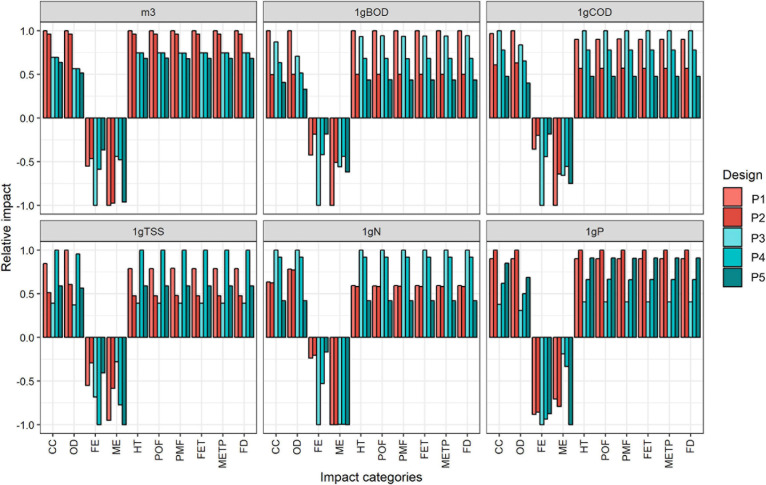
Comparative analysis of relative results of METland^®^ for several designs and periods.

The comparative results between designs and periods for different FU showed a wide variability (Figure 7). Our designed FU clearly evidenced that D2 was the foremost option. Nonetheless, the effectiveness of the treatments measured under different parameters obtained no direct correlation with the flow (characterized by our FU, m^3^). Therefore, the analysis of FU associated with the removal of pollutants (N, P, BOD, COD, and TSS) obtains a different trend among the periods. There is no remarkable difference between periods for every specific design. On the contrary, LCA results showed how the lower impact can shift among designs (e.g., BOD and COD removal from P5 at D2 and P2 at D1). Regarding N removal, P5 appeared as the most environmentally friendly option. In contrast, D1 (for both periods) showed similar results. ME and FE categories present a beneficial impact for all the FU due to the Net Environmental Balance perspective. Specifically, these results suggest that D1 could be more environmentally friendly under specific situations where high N removal is required. Nonetheless, a deeper analysis was required so a PCA was further conducted.

### Principal Component Analysis

**FIGURE 8:**
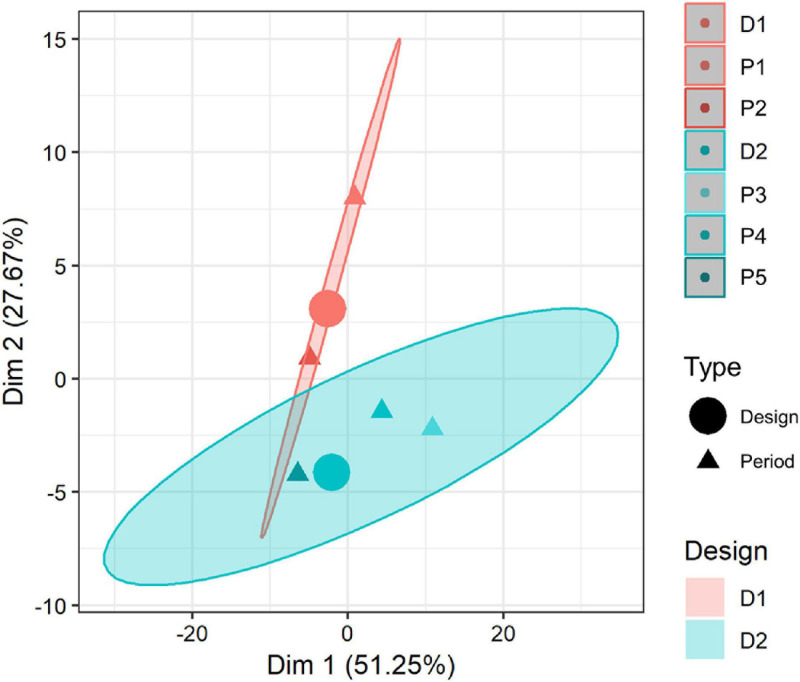
PCA distribution per design and period.

In order to delve into the impact of FUs on the different categories, a PCA was performed. Plotting all periods and designs (individuals) over the two most contributing dimensions revealed a variance of 77% (first dimension: 51.25% and second dimension: 27.52%) (Figure 8). Along such dimensions, the similarities between FUs, impact categories, and technical parameters could be easily compared. D1 periods were marked by Dim 2 and D2 periods by Dim 1. Furthermore, P2 results were similar to the P3–P5 periods.

**FIGURE 9:**
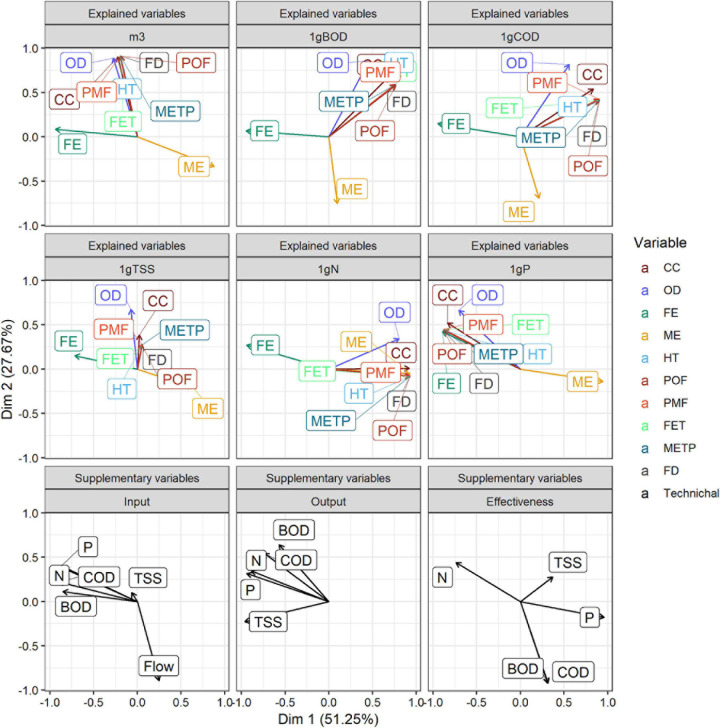
Results of PCA analysis for the results depending on the selected FU, category, and related to the technical parameters.

For a full interpretation of the PCA, the interaction among impact categories, evaluated FUs, and WW parameters (N, P, TSS, COD, BOD, and flow) were analyzed (Figure 9). Firstly, most of the impacts for each category (arrows) followed the same trend when 1 m^3^ was used as FU; indeed, they were opposite to the flow rate (input supplementary variable). Furthermore, our analysis revealed that the higher the volume treated, the lower the impact. However, both eutrophication categories, ME and FE, were dominated by the effectiveness of their key parameter (N and P, respectively). Such trends were maintained in the remaining FUs including all categories regarding N and P removal. Moreover, the most important trends were associated with the flow rate and N removal. In this sense, the aerobic configuration (D2) was correlated with the environmental foremost option within high flow rates of BOD and COD effectiveness. Nonetheless, aerobic–anoxic configuration (D1) could be more associated with adaptation to high N inlet. However, differences of the flow rates among periods were remarkable. Indeed, aerobic–anoxic configuration (D1) could be an option to consider for high N-content effluents with low flow rate or high N removal needs (e.g., manure effluents and small community effluents discharging to sensitive areas). Nonetheless, the limitations of the experimentation should be analyzed in further studies with a larger number of environmental factors such as meteorological conditions and climates to include dynamic LCA and perform a multi-scenario analysis to define precisely the frontiers between designs.

## Conclusion

Our study revealed that the new technology, so-called METland^®^, achieved environmental impacts as low as other nature-based solutions and significantly lower than those from conventional treatments like activated sludge. Furthermore, the solution is ideal for rural areas, considering the low energy requirements and high efficiencies to remove organic pollutants, nitrogen, and phosphates from urban wastewater.

Moreover, a combined technical and environmental analysis using a NEuB focus concluded that the aerobic downflow mode (D2) was the most environmentally friendly design, achieving the highest removal rates for carbon-based and nitrogen pollutants. The lower impact for such aerobic downflow configuration was also confirmed through a multi-functional analysis with different FU.

METland^®^ technology is being currently upgraded through innovation actions including the use of new materials and operation modes not included in the current study, so we can anticipate further that LCA analysis should be performed in the near future.

## Data Availability Statement

The raw data supporting the conclusions of this article will be made available by the authors, without undue reservation.

## Author Contributions

AE-N, JS-S, LP-A, and PL: conceptualization. AA-S and LP-A: data curation. JS-S and LP-A: formal analysis, methodology, investigation, software, and visualization. AE-N, EG-C, and JS: funding acquisition and supervision. AE-N, JS-S, and LP-A: writing—original draft. All authors contributed to the article and approved the submitted version.

## Conflict of Interest

The authors declare that the research was conducted in the absence of any commercial or financial relationships that could be construed as a potential conflict of interest.

## Footnotes

1 http://imetland.eu/

## Abbreviations

AEMET: Spanish Meteorological Agency
BOD_5,_: biochemical oxygen demand
CC: global warming potential
CENTA: Foundation Centre for New Water Technologies
COD: chemical oxygen demand
CW: constructed wetland
D1: first design
D2: second design
E: effectiveness
EC: electroconductive
ENEI: eutrophication net environmental impact
FD: fossil depletion potential
FE: freshwater eutrophication potential
FET: freshwater ecotoxicity potential
FU: functional unit
GHGs: green house gases
HT: human toxicity potential
I, inflow: influent
LCA: life cycle assessment
ME: marine eutrophication potential
MET: microbial electrochemical technologies
METP: marine ecotoxicity potential
MFC: microbial fuel cell
N: nitrogen
NEB: Net Environmental Balance
NEuB: net eutrophication balance
O, outflow: effluent
OD: ozone depletion potential
P: phosphorus
P1, first design: medium loading rate
P2, first design: high loading rate
P3, second design: low loading rate
P4, second design: medium loading rate
P5, second design: high loading rate
PCA: principal component analysis
PE: polyethylene
PMF: particulate matter formation potential
POF: photochemical oxidant formation potential
PVC: polyvinyl chloride
TN: total nitrogen
TP: total phosphorus
TSS: total suspended solids
WW: wastewater
WWT: wastewater treatment.
