# Supplementary material for: Assessing METland® Design and Performance Through LCA: Techno-Environmental Study With Multifunctional Unit Perspective
Source: Front Microbiol. 2021 Jun 11;12:652173. doi: 10.3389/fmicb.2021.652173 (PMC8226170; doi:10.3389/fmicb.2021.652173)
Supplement: Supplementary file 1 [file Data_Sheet_1.DOCX]

Supplementary Material

# Inventory tables.

|  | **Unit** | **Designs** | | | | | | |
| --- | --- | --- | --- | --- | --- | --- | --- | --- |
|  |  | **D1** | P1 | P2 | **D2** | P3 | P4 | P5 |
| **Operation** |  |  |  |  |  |  |  |  |
|  |  |  |  |  |  |  |  |  |
| **Inputs** |  |  |  |  |  |  |  |  |
| BOD_5_ | g·m^-3^ | 2.23E+02 | 1.61E+02 | 2.92E+02 | 1.81E+02 | 1.06E+02 | 1.46E+02 | 2.10E+02 |
| COD | g·m^-3^ | 4.67E+02 | 3.82E+02 | 5.59E+02 | 3.82E+02 | 2.31E+02 | 2.95E+02 | 4.44E+02 |
| TN | g·m^-3^ | 7.13E+01 | 6.73E+01 | 7.58E+01 | 6.07E+01 | 4.35E+01 | 4.83E+01 | 6.81E+01 |
| TP | g·m^-3^ | 8.70E+00 | 8.17E+00 | 9.29E+00 | 7.52E+00 | 5.93E+00 | 6.42E+00 | 8.20E+00 |
| TSS | g·m^-3^ | 1.61E+02 | 1.28E+02 | 1.96E+02 | 1.34E+02 | 1.78E+02 | 8.33E+01 | 1.27E+02 |
| Wastewater | m^3^·m^-3^ | 1.00E+00 | 1.00E+00 | 1.00E+00 | 1.00E+00 | 1.00E+00 | 1.00E+00 | 1.00E+00 |
| Solar energy | Mj m^-3^ | 1.81E-02 | 1.89E-02 | 1.61E-02 | 8.74E-03 | 9.10E-03 | 8.99E-03 | 8.30E-03 |
|  |  |  |  |  |  |  |  |  |
| **Outputs** |  |  |  |  |  |  |  |  |
| BOD_5_ | g·m^-3^ | 4.92E+01 | 4.01E+01 | 5.92E+01 | 1.80E+01 | 1.00E+01 | 1.37E+01 | 2.12E+01 |
| COD | g·m^-3^ | 1.20E+02 | 1.05E+02 | 1.36E+02 | 7.51E+01 | 4.44E+01 | 5.51E+01 | 8.79E+01 |
| TN | g·m^-3^ | 5.21E+01 | 4.78E+01 | 5.68E+01 | 4.52E+01 | 3.49E+01 | 3.90E+01 | 4.94E+01 |
| TP | g·m^-3^ | 7.30E+00 | 6.67E+00 | 7.99E+00 | 6.13E+00 | 3.46E+00 | 4.90E+00 | 7.18E+00 |
| TSS | g·m^-3^ | 2.32E+01 | 2.12E+01 | 2.54E+01 | 2.52E+01 | 1.59E+01 | 2.00E+01 | 2.89E+01 |
| Water emissions | m^3^·m^-3^ | 1.00E+00 | 1.00E+00 | 1.00E+00 | 1.00E+00 | 1.00E+00 | 1.00E+00 | 1.00E+00 |

Table A1. Life Cycle Inventory results for the operation phase. Design (D) and Period (P).

Table A2. Background processes from Ecoinvent database v3.4 used for the Life Cycle Assessment

| **Location** | **Process** |
| --- | --- |
| GLO | market for excavation, skid-steer loader \| excavation, skid-steer loader \| Cutoff, S |
| GLO | market for concrete, 20MPa \| concrete, 20MPa \| Cutoff, S |
| RoW | market for gravel, round \| gravel, round \| Cutoff, S |
| RoW | Pyrolized coke production, at plant, coking \| coke \| Cutoff, S |
| GLO | market for polyethylene, high density, granulate \| polyethylene, high density, granulate \| Cutoff, S |
| GLO | market for polyvinylidenchloride, granulate \| polyvinylidenchloride, granulate \| Cutoff, S |
| GLO | market for injection moulding \| injection moulding \| Cutoff, S |
| GLO | market for extrusion, plastic pipes \| extrusion, plastic pipes \| Cutoff, S |
| GLO | market for single-Si wafer, photovoltaic \| single-Si wafer, photovoltaic \| Cutoff, S |
